# Evaluation of tumor load in sentinel lymph node in patients with cutaneous melanoma

**DOI:** 10.1590/0100-6991e-20233521-en

**Published:** 2023-06-22

**Authors:** PEDRO DEAK DE ALMEIDA, LUCCAS LAVAREZE, CAROLINA EMERICK DA SILVA RANGEL, FERNANDA VIVIANE MARIANO, DIEGO VICTOR NUNES RODRIGUES, TIAGO ANTONIO BALDASSO, RENATO VENTURA FANNI, ANDRE LUIS MAION CASARIM, ANDRÉ DEL NEGRO, ALFIO JOSÉ TINCANI

**Affiliations:** 1 - Universidade Estadual de Campinas, Departamento de Cirurgia Geral - Área de Cabeça e Pescoço - Campinas - SP - Brasil; 2 - Universidade Estadual de Campinas, Departamento de Patologia - Campinas - SP - Brasil; 3 - Universidade Estadual de Campinas, Faculdade de Ciências Médicas - Curso de Medicina - Campinas - SP - Brasil

**Keywords:** Melanoma, Sentinel Lymph Node, Diagnosis, Melanoma, Linfonodo Sentinela, Diagnóstico

## Abstract

**Introduction:**

cutaneous melanoma (MC) is a malignant neoplasm derived from melanocytic cells with an aggressive behavior. It is usually associated with the multifactorial interaction of genetic susceptibility and environmental exposure, usually ultraviolet radiation. Despite advances in treatment, the disease remains relentless with poor prognosis. Sentinel lymph node (SLN) biopsy is a technique used to screen patients in need of lymph node dissection*.*

**Objectives:**

to correlate the tumor burden in the SLN with the mortality of patients undergoing SLN biopsy.

**Methodology:**

the medical records and histological slides of patients with MC who underwent SLN biopsy treated at HC-Unicamp from 2001 to 2021 were retrospectively analyzed. The positive SLN were measured according to the size of the tumor infiltration area, for analysis of the depth of invasion (DI), closest proximity to the capsule (CPC) and tumor burden (TB). For statistical analysis, associations between variables were analyzed using Fishers exact test, with post Bonferroni test and Wilcoxon test.

**Results:**

105 records of patients who underwent SLN biopsy of MC were identified. Of these, nine (8.6%) had positive SLN and 81 (77.1%) had negative SLN. The performed lymphadenectomies resulted in 55.6% (n=5) affected, 22.2% (n=2) without disease and 22.2% (n=2) were not performed. Mean CPC, TB, and DI were 0.14mm, 32.10mm and 2.33mm, respectively. Patients with T2 and T3 tumors were more likely to show the SLN affected (p=0.022). No patient with positive SLN died during follow-up.

**Conclusion:**

patients who presented T3 staging are the ones who most presented positive SLN.

## RESUMO

**Introdução:**

o melanoma cutâneo (MC) é uma neoplasia maligna de comportamento agressivo, derivada das células melanocíticas, geralmente causado pela associação de interação da suscetibilidade genética e a exposição ambiental. A biópsia do linfonodo sentinela (LNS) é um procedimento utilizado para rastreamento de doentes com necessidade ou não de linfadenectomia, diminuindo a exposição do paciente a cirurgias maiores.

**Objetivos:**

correlacionar a carga do tumor no LNS com a mortalidade de pacientes com MC.

**Métodos:**

foram examinados retrospectivamente prontuários e lâminas histológicas de doentes com MC submetidos a biópsia de LNS atendidos no HC-Unicamp entre o período compreendido de 2001 a 2021. Os LNS positivos foram mensurados quanto ao tamanho da área de infiltração do tumor, para análise da profundidade de invasão (PI), menor proximidade com a cápsula (MPC) e carga do tumor (CT). As associações entre as variáveis foram analisadas pelo teste Exato de Fisher, com pós teste de Bonferroni e Wilcoxon.

**Resultados:**

foram identificados 105 pacientes com biópsia de LNS, sendo nove (8,6%) casos com LNS positivo. A média de MDC, CT e PI foram 0,14 mm, 32,10 mm, e 2,33 mm, respectivamente. Doentes com tumores T2 e T3 apresentaram maior chance de acometimento do LNS (p=0,022). Nenhum paciente com LNS positivo foi a óbito durante o acompanhamento.

**Conclusão:**

doentes que apresentaram tumores maiores (T2 e T3) apresentaram maior chance de linfonodos sentinelas positivos. Além do tamanho do tumor, a presença de maior carga do tumor demonstrou que os mesmos podem ser beneficiados pela biópsia de LNS.

## INTRODUCTION

Cutaneous melanoma is a malignant neoplasm that is often aggressive and caused by melanocytic cells, responsible for producing melanin, the substance that determines skin color^1^. The lesion is multifactorial, being caused by the interaction between genetic susceptibility and environmental exposure, such as mainly sun exposure, artificial tanning, fair skin, increased number of melanocytic and/or dysplastic nevi, freckles, and family history of the disease^2^ ^,^ ^3^. Melanoma is estimated to constitute less than 5% of skin cancers, but it is responsible for about 95% of deaths from cancer in this organ, being the most severe skin cancer^5^. In Brazil, data from the National Cancer Institute for the year 2020 indicated melanoma incidence rates of 4.03 new cases per 100,000 in males and 3.94 new cases per 100,000 in females^4^ ^,^ ^5^. The main protective factor for the reduction of mortality is early diagnosis, the prognosis being directly proportional to the invasion in the depth of the skin^6^.

Sentinel lymph node (SLN) biopsy is a technique that aims to avoid aggressive lymphadenectomy surgeries that can lead to limiting complications for patients, such as limb lymphedema and neurovascular lesions. Conceptually, the technique consists of marking the SLN with lymphoscintigraphy prior to surgery, and intraoperatively locating it with a portable gamma radiation detector. Subsequently, the SLN is analyzed by the pathologist for the presence of metastasis. Once negative, the ganglionic chain can be considered free of metastasis and would avoid additional procedures^7^, being considered of high relevance for risk assessment and treatment strategy^7^. In this study, we aimed to correlate tumor load with mortality in patients undergoing SLN biopsy.

## METHODS

This is a retrospective study with analysis of medical records and histological slides of patients who underwent SLN biopsy in melanoma treated at the Discipline of Head and Neck Surgery at UNICAMP Clinics Hospital from January 2001 to January 2021, after approval from the Ethics in Research Committee of the State University of Campinas (UNICAMP), under CAAE number 59360322.1.0000.5404. We included cases that had a slide stained with Hematoxylin and Eosin (H&E) with sufficient tissue for histopathological evaluation and subsequent analysis. We excluded patients who had previously been treated or manipulated in the SLN drainage area.

In the analysis of medical records, we collected data regarding age, color, race or ethnicity, sex, primary location of the melanoma, Breslow index of the primary melanoma, T staging of the primary melanoma, and survival. We divided patients into positive and negative SLN groups for the analyses.

**Figure 1:**
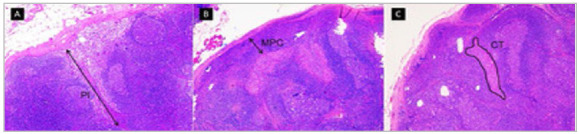
DI, CPC, and TB measurement methodology.

Two examiners analyzed the H&E slides with their respective diagnoses to confirm the presence or absence of metastasis in the sentinel lymph nodes. Photomicrographs of positive SLNs were taken using five- and ten-fold augmentation lenses, according to the size of the tumor infiltration area, for analysis of depth of invasion (DI), closest proximity to the capsule (CPC), and tumor burden (TB). The photomicrographs were taken using a Leica^®^ microscope (Leica Microsystems, Switzerland) and processed using the Leica^®^ software, version 4.2.0 (Leica Microsystems, Switzerland). We defined DI as the largest area of melanoma infiltration in millimeters (mm) inside the lymph node (Figure 1A). The CPC corresponded to the shortest distance (mm) between the tumor and the inner portion of the lymph node capsule (Figure 1B). Finally, TB was the sum of all metastatic areas in square millimeters (mm^2^) in the lymph nodes (Figure 1C). Cases with more than one metastasis focus were photographed and evaluated in several fields, with the tabulation of the value corresponding to the definition of its variable (DI or CPC). We analyzed the images in the ImageJ software (National Institutes of Health, USA), version 1.53v, using a millimeter ruler photographed in the 5x or 10x lenses for standardization.

For statistical analysis, we assessed associations between variables using the Fisher’s exact test, with Bonferroni post-test and Wilcoxon test. All analyzes were performed using the R software (R Foundation for Statistical Computing, Vienna, Austria) with a significance level of 5%. 

## RESULTS

We identified 105 records of patients who underwent a melanoma SLN biopsy in the study period. This number was reduced because in the period between 2004 and 2006 this procedure was not performed due to operational problems. Sample demographics are described in Table 1. The sample had a mean age of 54.1 ± 14.0, and a similar distribution between sexes, 50.5% (n=53) women and 49.5% (n=52) men. Most patients were white (94.3%; n=99), with tumors on the trunk (38%, n=40) and upper limbs (30.4%, n=32). As for the distribution of the tumor by sex, four (7.5%) cases were found in women in the head and neck, in contrast to seven (13.5%) in men. In the trunk, we identified 20 cases (37.7%) in women and 20 (38.4%) in men. We found Melanoma in the upper limbs in nine (16.9%) female cases and in 13 (25%) males. Involvement in the lower limbs happened in 20 (37.7%) women and in 12 (23%) men.

**Table 1:** Demographic characteristics of the sample, 2022.

Variable	n=105
Age on procedure date, mean (SD)	54.1 (14.0)
Sex, n (%)	
Female	53 (50.5)
Male	52 (49.5)
Color, n (%)	
White	99 (94.3)
Black	2 (1.9)
Brown	3 (2.9)
Not informed	1 (0.9)

**Figure 2:**
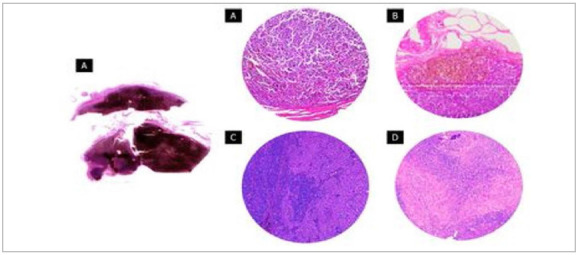
A) SLN almost entirely involved by cutaneous melanoma metastasis. Metastasis can occur in scattered cells (B) or in a small nest of cells in close contact with the capsule (C). Metastases from amelanotic melanoma (D and E) forming nests of malignant cells in the SLN parenchyma.

Table 2 presents the characteristics of the SLN-positive and SLN-negative groups. There were nine (8.6%) patients with positive SLN (Figure 2) and 81 (77.1%) with negative SLN. We could not find this information for 15 individuals (14.3%). Patients with more advanced tumors (T2 and T3) had a greater chance of SLN involvement (p=0.022).

**Tabela 2:** Sample characteristics according to the presence of positive SLN.

Variables	positive SLN (n=9)	negative SLN (n=81)	p -value
Age on procedure date, mean (SD)	57.70 (13.6)	53.80 (14.0)	0.541
Sex, n (%)			
Female	6 (66.7)	37 (45.7)	0.301
Male	3 (33.3)	44 (54.3)	
Color, n (%)			
White	9 (100.0)	76 (93.8)	1,000
Black	-	2 (2.4)	
Brown	-	3 (3.7)	
Breslow ‡, mean (sd)	2.13 (0.7)	2.07 (1.7)	0.152
Location			
Head	-	11 (13.6)	0.650
Trunk	3 (33.3)	32 (33.3)	
Lower limbs	4 (44.4)	22 (27.2)	
Upper limbs	2 (22.2)	16 (19.8)	
Staging T †, n (%)			
T1	1 (14.3)	12 (16.7)	0.022
T2*	1 (14.3)	41 (56.9)	
T3*	5 (71.4)	13 (18.1)	
T4	-	6 (8.3)	
Ulceration †, n (%)			
Present	3 (37.5)	26 (36.6)	1,000
Absent	5 (62.5)	45 (63.4)	
Variables	positive SLN (n=9)	negative SLN (n=81)	p -value
TL (mm³), mean (sd)	32.10 (95.1)	-	-
CPC (mm), mean (sd)	0.14 (0.3)	-	-
DI (mm), mean (sd)	2.33 (3.6)	-	-
Status on last follow-up			
Alive	9 (100.0)	80 (98.8)	1,000
Dead	-	1 (1.2)	

^*‡*^ n=79: 7 positive SLN; 72 negative SLN; † n=79; * Statistical difference between groups; DI: depth of invasion; CPC: closest proximity to the capsule; TB: tumor burden.

The histological information of the nine patients with positive SLN are shown in Table 3. Most had tumors in close contact with the capsule (n=6), resulting in 0 mm CPC (Figure 2C). The mean TB was 32.10 ± 95.1mm². This value was increased due to a SLN completely affected by the disease (patient 5). The mean DI was 2.33 ± 3.6mm.

**Table 3:** Histological measurements of positive SLN.

Patient	CPC (mm)	TB (mm²)	DI (mm)
1	0	0.591	0.464
2	0.027	0.031	5,743
3	0	0.313	0.48
4	0.563	0.772	1.605
5	0	285.67	10.64
6	0	0.829	1.023
7	0	0.015	0.113
8	0.63	0.183	0.834
9	0	0.237	0.042

*DI: depth of invasion; CPC: closest proximity to the capsule; TB: tumor burden.*

The performed lymphadenectomies resulted in 55.6% (n=5) affected, 22.2% (n=2) without disease, and 22.2% (n=2) were not performed. The five-year survival of the overall sample was 92.4% and the 10-year survival rate was 96.1%. However, 29.3% (n=29) of patients were lost to follow-up in the last three years, in addition to 5.7% of patients’ records not showing any follow-up. In the group of patients with positive SLN, there was a 44% loss to follow-up, with a mean survival until the last visit of 2.2 years. The average survival of the followed patients was 10.79 years.

## DISCUSSION

Cutaneous melanoma (CM) is an aggressive malignant neoplasm that can have an unfavorable clinical course when diagnosed in late stages^2^ ^,^ ^3^. Although early diagnosis is the best way to improve prognosis, treatment for this tumor is still challenging^6^. Despite advances in drug therapy with BRAF inhibitors^8^, surgery is still the primary treatment for tumors and advances in the technique are needed to reduce morbidity and recurrence. We highlight the SLN biopsy as a procedure that encompasses the early diagnosis of lymph node metastases, which can avoid mutilating surgeries.

SLN biopsy has been used in several types of cancer with the objective of tracking occult metastases, with important results in prognosis. In melanoma, SLN positivity helps in the stratification of intermediate and high risk patients, identifying the ones who will benefit from adjuvant therapies^9^. The recommendation for SLN biopsy is made for patients without clinical evidence of metastasis in lymph nodes and who have tumors thicker than 1mm or with lesser thickness and present ulceration, since the frequency of SLN positivity in melanomas is usually related to these findings^28,29^. However, it is important to point out that tumors smaller than 1mm have a probability of approximately 8.4% of a positive SLN^30^ and other clinical factors such as ulceration and tumor thickness must be taken into account when deciding on a SLN biopsy^10^ ^,^ ^11^. Our findings indicate that larger tumors (T2 and T3) may present an increased chance of positive SLN, which may compose the clinical factors that indicate the procedure. Corroborating with the data found, the prevalence of positive SLN in cutaneous melanomas increased according to the size of the primary tumor, and the most advanced tumors displayed 17% to 29% positivity^12^.

In this sample, a reduced number of patients had a positive SLN (9%). In the literature, the general positivity rate ranges from 12% to 26%^13^ ^-^ ^15^. In a study carried out with 682 patients, the SLN positivity rate was 12.9% for tumors with a thickness of less than 2mm^35^. One of the fundamentals of the SLN biopsy is that most patients with disease in the lymph nodes are limited to the SLN, but studies show that a small number of patients prove this fact. Faries et al. (2017) pointed out that although total lymph node dissection leads to greater regional disease control, it does not impact disease-specific survival in patients with SLN metastases^16^ ^,^ ^17^.

Therapeutic decisions regarding the positivity of an SLN are still discussed in the literature. The absence of standardization and criteria to quantify the tumor invasion load in SLN makes it difficult to define a methodology that points to a positive SLN at risk. The Rotterdan criterion states that the larger the TB, the greater the chance of involvement of the ganglionic chain, and SLNs with TB greater than 1mm could benefit from a complete dissection^18^ ^,^ ^19^. However, it is not uncommon for other methodologies to be found in the literature. Despite the heterogeneity of methods and results, there is a consensus that the chance of involvement of non-sentinel lymph nodes is proportional to the size of the TB in the SLN^20^ ^,^ ^21^.

Evidences point out that patients submitted to SLN biopsy present a better survival than those who remained under lymph node surveillance^11^. Nevertheless, later studies with similar methodology did not indicate difference in the prognosis of patients with melanoma, regardless of SLN management^17^. Patients with lymph nodes showing extracapsular or microsatellite extension or more than three involved lymph nodes generally do comprise these studies’ samples, in fact belonging to higher risk groups and generally requiring additional therapeutic approaches. The present study was unable to determine the safety of avoiding complete lymph node dissection in patients who do not undergo frequent medical evaluations or who cannot undergo lymph node imaging in their follow-up^16^. Studies indicate that patients with cutaneous melanoma and positive SLN have a mean survival of 10 years, varying according to clinical staging, but longer than metastases in non-sentinel lymph nodes^22^. In the present study, there were no deaths in the group with positive SLN, precluding additional survival analyses. A limitation of this work is in patients’ follow-up. Although many patients return for routine appointments at the unit where they were treated, a portion is referred to the primary health sector, making it impossible to collect information after a period of time. A second limitation is mortality. Although our death records come from two sources (medical records and cancer death records), both data have geographic limitations. While the former would require this information to be reported herein, the latter only includes patients who died in the state of São Paulo. Additional works that contemplate a deep research in the patient’s outcome are necessary for the understanding of the role of the SLN biopsy in the prognosis of patients with cutaneous melanoma.

## CONCLUSION

Patients who had larger tumors (T2 and T3) were more likely to have positive sentinel lymph nodes. In addition to tumor size, the presence of greater tumor load demonstrated that patients could benefit from SLN biopsy. We could confirm the impact of positive or negative SLN on patient survival due to limitations in data collection.

## References

[1] Ahmed B, Qadir MI, Ghafoor S (2020). Malignant Melanoma Skin CancerDiagnosis, Prevention, and Treatment. Crit Rev Eukaryot Gene Expr.

[2] Elwood JM, Jopson J (1997). Melanoma and sun exposure An overview of published studies. Int J Cancer.

[3] Lodde G, Zimmer L, Livingstone E (2020). Malignes Melanom. Pathologe.

[4] Hayes AJ, Maynard L, Coombes G (2016). Wide versus narrow excision margins for high-risk, primary cutaneous melanomas long-term follow-up of survival in a randomized trial. Lancet Oncol.

[5] Nader MG, Munhoz RR, Teixeira MLP (2020). Trends in Melanoma Mortality in Brazil A Registry-Based Study. JCO Glob Oncol.

[6] Carr S, Smith C, Wernberg J (2020). Epidemiology and Risk Factors of Melanoma. Surg Clin North Am.

[7] Schadendorf D, van Akkooi ACJ, Berking C (2018). Melanoma. Lancet.

[8] Corrie P, Meyer N, Berardi R (2022). Comparative efficacy and safety of targeted therapies for BRAF-mutant unresectable or metastatic melanoma Results from a systematic literature review and a network meta-analysis. Cancer Treat Rev.

[9] Carr MJ, Monzon FA, Zager JS (2022). Sentinel lymph node biopsy in melanoma beyond histologic factors. Clin Exp Metastasis.

[10] Gershenwald JE, Thompson W, Mansfield PF (1999). Multi-Institutional Melanoma Lymphatic Mapping Experience The Prognostic Value of Sentinel Lymph Node Status in 612 Stage I or II Melanoma Patients. J Clin Oncol.

[11] Morton DL, Thompson JF, Cochran AJ (2014). Final Trial Report of SentinelNode Biopsy versus Nodal Observation in Melanoma. N Engl J Med.

[12] van der Ploeg APT, van Akkooi ACJ, Rutkowski P (2011). Prognosis in Patients With Sentinel Node-Positive Melanoma Is Accurately Defined by the Combined Rotterdam Tumor Load and Dewar Topography Criteria. J Clin Oncol.

[13] Woods JFC, De Marchi JA, Lowery AJ (2015). Validation of a nomogram predicting sentinel lymph node status in melanoma in an Irish population. Ir J Med Sci.

[14] Bleicher RJ, Essner R, Foshag LJ (2003). Role of Sentinel Lymphadenectomy in Thin Invasive Cutaneous Melanomas. J Clin Oncol.

[15] Ranieri JM, Wagner JD, Wenck S (2006). The Prognostic Importance of Sentinel Lymph Node Biopsy in Thin Melanoma. Ann Surg Oncol.

[16] Faries MB, Thompson JF, Cochran AJ (2017). Completion Dissection or Observation for Sentinel-Node Metastasis in Melanoma. N Engl J Med.

[17] Angeles C V, Kang R, Shirai K (2019). Meta-analysis of completion lymph node dissection in sentinel lymph node-positive melanoma. Br J Surg.

[18] van Akkooi ACJ, de Wilt JHW, Verhoef C (2006). Clinical relevance of melanoma micrometastases (&lt;0 1mm) in sentinel nodes: are these nodes to be considered negative?. Ann Oncol.

[19] van Akkooi ACJ, Nowecki ZI, Voit C (2008). Sentinel Node Tumor Burden According to the Rotterdam Criteria Is the Most Important Prognostic Factor for Survival in Melanoma Patients. Ann Surg.

[20] Morrison S, Han D (2021). Re-evaluation of Sentinel Lymph Node Biopsy for Melanoma. Curr Treat Options Oncol.

[21] Nguyen CL, McClay EF, Cole DJ (2001). Melanoma thickness and histology predict sentinel lymph node status. Am J Surg.

[22] Keung EZ, Gershenwald JE (2018). The eighth edition American Joint Committee on Cancer (AJCC) melanoma staging system implications for melanoma treatment and care. Expert Rev Anticancer Ther.

